# Histone methyltransferase SETD2: a potential tumor suppressor in solid cancers

**DOI:** 10.7150/jca.38391

**Published:** 2020-03-05

**Authors:** Rui Chen, Wei-qing Zhao, Cheng Fang, Xin Yang, Mei Ji

**Affiliations:** Department of Oncology, the Third Affiliated Hospital of Soochow University, The First People's Hospital of Changzhou, No. 185 Juqian Road, Tianning District, Changzhou 213003, China.

**Keywords:** SETD2, Mutation, Tumor suppressor, Solid cancers

## Abstract

Epigenetic regulation plays an important role in the occurrence, development and treatment of malignant tumors; and a great deal of attention has been paid to the histone methylation level in recent years. As a 230-kD epigenetic regulator, the histone H3 lysine 36 histone (H3K36) methyltransferase SETD2 is a key enzyme of the nuclear receptor SET domain-containing (NSD) family, which is associated with a specific hyperphosphorylated domain, a large subunit of RNA polymerase II (RNAPII), named RNAPII subunit B1 (RPB1), and SETD2 which methylates the ly-36 position of dimethylated histone H3 (H3K36me2) to generate trimethylated H3K36 (H3K36me3). SETD2 is involved in various cellular processes, including transcriptional regulation, DNA damage repair, non-histone protein-related functions and some other processes. Great efforts of high-throughput sequencing have revealed that SETD2 is mutated or its function is lost in a range of solid cancers, including renal cancer, gastrointestinal cancer, lung cancer, pancreatic cancer, osteosarcoma, and so on. Mutation, or functional loss, of the SETD2 gene produces dysfunction in corresponding tumor tissue proteins, leading to tumorigenesis, progression, chemotherapy resistance, and unfavorable prognosis, suggesting that SETD2 possibly acts as a tumor suppressor. However, its underlying mechanism remains largely unexplored. In the present study, we summarized the latest advances of effects of SETD2 expression at the mRNA and protein levels in solid cancers, and its potential molecular and cellular functions as well as clinical applications were also reviewed.

## Introduction

As a process consisting of complex and consecutive changes with high morbidity and mortality, cancer is the leading cause of death in the world 1. Recent studies have revealed that histone methylation plays a crucial role in regulatory mechanism, histone lysine methyltransferases (KMTs) are associated with cell biosynthesis and its gene mutation or functional loss as well as subsequent downstream signaling pathways facilitates oncogenic processes 2. Histone methyltransferase SETD2 (also known as HYPB) is first isolated from human hematopoietic stem cells, and it is thought to be associated with Huntington's disease 3. Previous studies have reported that many other KMTs can catalyze H3K36 to generate monomethylated histone H3 (H3K36me1) or H3K36me2, such as ASH1L (absent small and homeotic disks protein 1 homolog), NSD1, NSD2, NSD3 (nuclear receptor-binding SET domain-containing proteins 1-3) and SMYD2 (SET and MYND domain containing 2). SETD2 is a key member of nuclear receptor SET domain-containing (NSD) family 4, and it is the only methyltransferase which can alter the trimethylation status of H3K36 and regulate protein structure as well as its function 5,6. SETD2 is mutated or its function is lost in various solid tumors 7,8, leading to imbalance in methylation, demethylation and epimutation, which eventually causes tumorigenesis. Loss of SETD2 affects the progress of the transcriptional elongation, resulting in failure of DNA damage repair. SETD2 deficiency has also been linked to p53, downstream signaling pathway and non-histone protein process. All of these data suggest that mutation of SETD2 gene or its functional deficiency exists in tumors, and it may function as a tumor suppressor (Table 1).

## Protein structure of SETD2

We denominated SETD2 with three genes, Su (var)3-9, enhancer of zeste and trithorax. SETD2 is located at cytogentic band p21.31 of chromosome 3, and SETD2 protein consists of three main functional domains as follows: (1) the methyltransferase activity domains: AWS (associated with SET), SET and PS (post-SET); (2) protein-binding domains: WW (tryptophan-tryptophan), CC (Coiled-Coiled) 9,10 and SRI (Set2-Rpb1 interacting); and (3) other unclear domains.

## The biological function and potential mechanism of SETD2

Previous studies have identified that SETD2 and its dependent H3K36me3 both participate in a series of cellular processes 11. Mutation of SETD2 gene and dysfunction of downstream signaling pathways affect biological functions in many different ways, eventually causing tumorigenesis. However, the underlying mechanism remains unknown. In the present study, we summarized the latest research advances in terms of potential cellular and molecular mechanisms.

### Transcriptional regulation

Transcription is a highly regulated and congenitally stochastic biochemical process 12, and such process is carried out by binding the specific regions of DNA and RNA 13. Transcriptional regulation is achieved by varying the rates of different transcription procedures 14. The methyltransferase Set2 in yeast, which has homology similarity to human SETD2 9, is responsible for histone methylation, and both of Set2 and H3K36me3 are involved in transcription 15-17. H3K36me3 can prevent initiation of spurious transcription by recruiting histone deacetylase complexes, and depletion of Set2 results in increased level of this adverse transcription 16, 18-20. Set2 also plays a role in elongation through directly associating with RNAPII via WW domain 21, 22. Consistently, SETD2 and H3K36me3 participate in transcriptional program in humans as well 15, 23-25. SETD2 binds to the hyperphosphorylated carboxy-terminal domain (CTD) of RNAPII via the SRI domain, and then recruits MORF-related gene on chromosome 15 (MRG15) and polypyrimidine tract binding protein (PTB) during transcriptional process 26.

### DNA damage repair

DNA is a complex molecule that stores genetic information packaged inside histones. DNA damage can be triggered by various causes, and failure of damage repair is lethal for genome stability, leading to the occurrence of malignant diseases. DNA double-strand break (DSB) is one form of DNA damage, and its misrepair is a feature of tumors 27. SETD2 and H3K36me3 are mainly related to human homologous recombination (HR) 28, and the latter is a critical repair as well as tolerance pathway 29, which facilitates DNA DSB repair 30. SETD2 medicates the binding of H3K36me3 to LEDGF (lens epithelium-derived growth factor)/CtIP (C-terminal binding protein interacting protein) complex 31,32 by promoting DSB resection, recruiting DNA-repair members, including RAD51 and replication protein A (RPA) to damaged sites, and then completing repair-related events 33. In renal cancer, SETD2 mutation leads to unrepairable DNA damage, by which the key suppressor p53-mediated checkpoint can't be activated 30. DNA mismatch repair (MMR) is known as a biological pathway which ensures genome stability 34. SETD2 and H3K36me3 participate in human MMR in vivo 35, 36, H3K36me3 is required to recruit MMR protein hMutSα (hMSH2-hMSH6) (a significant player in MMR-associated processes) by direct combination with the specific MSH6's conserved domain PWWP (proline-tryptophan-tryptophan-proline) 37-39, and then hMutSα is located to chromatin. Further studies have found that SETD2 and H3K36me3 neither physically modulate MMR nor alter the expression level or function of MMR gene 36.

### Other functions

**Figure 1:**
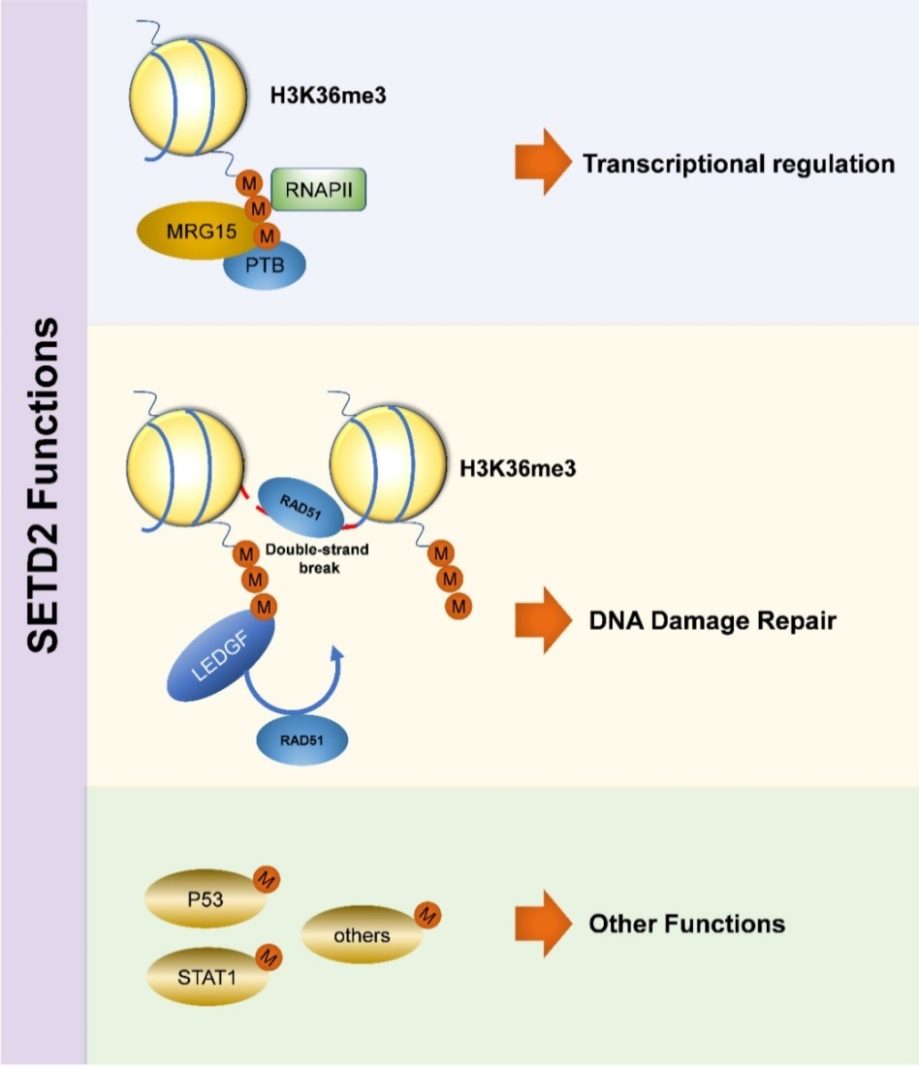
Biological Function of SETD2

Interestingly, the biological function of SETD2 is more than just that. SETD2 has been described to be involved in the process of tumorigenesis through combining tumor suppressor p53 and regulating its downstream genes 40, the WW and SET domains are necessary during this process 41, p53 is a key transcriptional activator which regulates cell cycle and apoptosis, and SETD2 down-regulates the expression of downstream hdm2, which is a p53 target, leading to enhanced p53 protein stability 41. SETD2 also has some functions with regard to non-histone targets, and its levels are significantly increased during mitosis, wherein cytoplasmic methylation of tubulin by SETD2 maintains the genome stability by promoting proper chromosome segregation 42. Recently, SETD2 protein has been identified to interact with STAT1 (signal transducers and activators of transcription), finally causing dysfunction of cell signaling pathway 43. Besides, SETD2 and H3K36me3 are implicated in alternative splicing (AS) 44,45, and this event is related to histone modification 46-49. H3K36me3 is also related to crosstalk 11 and genomic integrity (Figure 1) 33.

## The medical application of SETD2 in solid cancers

### SETD2 in reproductive and urinary system cancers

#### SETD2 in renal cancer

Studies have identified that 3P chromosome loss occurs in almost all cases of clear cell renal cell cancer (ccRCC) 50-53, causing mutation of multifarious tumor suppressor genes either independently or simultaneously 54. Some diseases are emerging with poor prognosis 55-57, and the therapeutic resistance has gradually developed. SETD2 mutation fluently occurs in renal cancer 11, 58-62, and the absence of SETD2 protein is more evident than deficiency of the SETD2 gene itself. The mutation of SETD2 gene in ccRCC has a proportion of 34.07%, with such a mutation being related to producing an aggressive cancer phenotype 63. The expression levels of SETD2 and H3K36me3 is associated with the tumor size, clinical stage and risk of carcinoma-related death, suggesting the worse prognosis in ccRCC patients with or without metastasis 57,64,65. Besides, a comprehendsive evaluation of SETD2 and H3K36me3 expressions may be a factor in predicting preoperative risk stratification and guiding future treatment in patients with early-stage ccRCC 57. Mechanically, SETD2 in renal cancer plays a role in epigenetic dysfunction and metabolism regulation. H3K36me3 works with H4K16ac to promote transcriptional activation 11, and a lower level of H3K36me3 fails to accomplish DNA repair and AS 45, and activate p53 30 in renal cancer. SETD2 deficiency is associated with metabolism of three major substances, including carbohydrate, glycosaminoglycan and creatine 11, and SETD2-inactive cells are likely to enhance the PGC1α expression at the protein (PGC1α has been known as a stimulator in tumor of digestive system^66^) level and undergo a higher level of tricarboxylic acid cycle process with released energy. The dysfunction of SETD2-PGC1α metabolic pathway may act as a stimulating factor in ccRCC and provide a potential based on metabolomics of targeted treatment 67. SETD2 is a leading cause of sunitinib resistance and may be related to MCL-1 protein expression 68. All of these studies have indicated that SETD2 acts as a tumor suppressor in renal cancer 69.

#### SETD2 in prostate cancer

Global cancer statistics have shown that prostate cancer is the second most commonly diagnosed malignancy in male patients 1,70,71. The most appropriate treatment approach is chosen according to the pathological type, clinical stage and molecular subtype. In combination with The Cancer Genome Atlas (TCGA), correlation and regression analysis show that histone methyltransferase plays an important role in prostate cancer and may be linked to genesis and progression of disease. SETD2 mutation does exist in human prostate cancer. Surprisingly, it is significantly clustered in prostate cancer samples over-expressing androgen receptors, indicating that functional loss of SETD2 may lead to resistance to surgical and drug castration in prostate patients and the specific underlying mechanism needs to be further explored 72.

### SETD2 in digestive system cancers

#### SETD2 in gastric cancer (GC)

Studies have identified that mutation of SETD2 gene or its functional loss also occurs in advanced GC 73-75. The SETD2 expression at the mRNA and protein levels has been respectively investigated by immunohistochemistry, quantitative PCR (qPCR), real-time PCR, and immunoblotting. The results have shown that the SETD2 expression at the mRNA and protein levels is remarkably lower in tumor tissue compared with adjacent normal tissue, and the SETD2 expression at the mRNA level is declined in nearly 80% tumor tissue samples. Besides, the expression level of SETD2 is significantly linked to tumor size, TNM stage, and lymph node metastasis, and down-regulated SETD2 expression is significantly associated with lower overall survival and lower 5-year survival in GC patients. All of these data have identified that SETD2 may be one negative prognosis factor of advanced GC patients 73,74. A univariate analysis has discovered that SETD2 mutation or its functional loss in GC patients in Singapore, especially in high-risk gastrointestinal stromal tumor (GIST) 75. Further studies have identified that in GC cell lines, over-expression of SETD2 can significantly inhibit the biological functions, including cell proliferation, migration and invasion 73. SETD2 may act as a tumor suppressor gene, and the specific mechanism is divided into two aspects. SETD2 binds to p53 to form a co-immunoprecipitate, and this combination can occur under various conditions. Moreover, SETD2 can affect p53 and then participate in regulating activity of its transcriptional factors 73. Loss of SETD2 subsequently leads to a lower level of SETD2-dependent H3K36me3, and SETD2 silenced cells show a tendency to promote DNA damage. Therefore, SETD2 may act as a suppressor gene and have the potential to serve as a molecular marker for prognosis and treatment in GC and predict the GIST patient risk stratification.

#### SETD2 in colorectal cancer (CRC)

Recently, SETD2 has been clarified to play a suppressive role in CRC 76, and inactivation of SETD2 induces tumor malignant potential and increases susceptibility to tumorigenesis. Further studies on the mechanism of SETD2 have shown that SETD2 protects the intestinal cells via fine-tune Wnt signaling pathway and some other pathways, such as SIRT7, JAG1 and PKM2. SETD2 deficiency may promote the expression of dishevelled segment polarity protein 2 (DVL2), resulting in increased stabilization and transcriptional activity of β-catenin, leading to promoted oncogenesis, proliferation and metastasis. In addition, functional loss of SETD2 disturbs SETD2-H3K36me3 expression and eventually affects AS in CRC 77, and such dysfunction is also linked to intestinal tumorigenesis.

#### SETD in pancreatic cancer

Recent studies have found that epigenetic mechanisms also play a role in pancreatic cancer. SETD2 gene alters or mutates in around 10% patients with pancreatic cancer, and such data are consistent with TCGA 8,78. Studies have analyzed various clinical stages and pathological types of pancreatic cancer, mainly composed of normal pancreatic tissues, pancreatic intraepithelial neoplasia (PanIN), pancreatic ductal adenocarcinoma (PDA), intraductal papillary mucinous neoplasm (IPMN) and advanced PDA, and the results show that the expression of SETD2 in pancreatic cancer tissues is significantly decreased compared with the normal tissues. No matter it is precancerous lesions or advanced metastatic cancer tissue, the difference is statistically significant. More interestingly, the lowest SETD2 expression is found in PanIN lesions, supporting that SETD2 is associated with the early development and metastasis in pancreatic cancer. In addition, unlike other tumors, bioinformatic analysis indicates that there is no significant correlation between the SETD2 expression and prognosis in pancreatic cancer patients 79.

### SETD2 in lung cancer

Lung cancer is one of the world's most pressing diseases, which is characterized by high morbidity and poor survival 1,80. With the development of targeted therapy, anti-angiogenenic therapy and immunotherapy have gradually been matured, leading to effectively improved survival rate. Mutation of SETD2 gene or its functional loss in lung cancer 81-86 has a higher frequency in metastatic sites compared with primary sites, which is linked to poor prognosis 82,83. SETD2-deficient cells display a robust tumor-driver property 41, functional loss of SETD2 and lower expression of H3K36me3 accelerate the progression of both early- and late-stage lung adenocarcinoma (LUAD), and such situation may be associated with deficiency of SWI/SNF complex 87. Previous reports have identified that SETD2 deficiency is involved in resistance of chemotherapy drugs in solid tumors 68,72, and SETD2 also plays a key role in the treatment of lung cancer. Cisplatin constitutes a mainstay drug in combination chemotherapy regiments with other drugs in treatment of advanced non-small cell lung cancer (NSCLC), SETD2 mutation facilitates acquired cisplatin resistance due to decreased apoptosis of cancer cells, and such apoptosis occurs with abnormal H3K36me3 expression and ERK signaling pathway 82, finally contributing to worse relapse-free survival 88, 89. In conclusion, SETD2 may function as a tumor suppressor gene in the development of lung cancer.

### SETD2 in other tumors

Previous research has shown that mutations of SETD2 gene frequently occur in high-grade gliomas and only occur in the area named cerebral hemispheres, and such situation is more common in children and adolescents than elderly populations. Functional loss of SETD2 and deficiency of H3K36me3 may be linked to tumorigenesis 90. Interestingly, SETD2 mutation also occurs in central nervous system tumors, including low-grade gliomas and non-glial tumors, and this gene mutation also occurs in people over 55 years of age 91, 95, 96. According to the TCGA and METABRIC databases, SETD2 is mutated in all subtypes of breast cancer with a proportion of 2.62%, and its incidence in triple-negative breast cancer is 1.2%. However, it remains unclear whether SETD2 is mutated in Luminal B subtype, Luminal A and Her+. Further studies have shown that in all types of breast cancer, the expression level of SETD2 is significantly related to the prognosis of patients and that the higher the expression, the better the prognosis. Such correlation also exists in the triple-negative breast cancer, and the difference is more obvious, indicating that SETD2 mutation is linked to poor prognosis. However, the SETD mutation has few influences on patient outcome with chemotherapy 94. Another study, which is the first one in the literature, also identified that the expression of SETD2 at the mRNA level in malignant tissue is markedly reduced compared to normal tissue. Further detection has shown that the expression of SETD2 is negatively correlated with tumor grade, stages and lymph node metastasis. These two studies have confirmed that SETD2 may play a role in tumor suppression, and it can be used as a prognostic marker in breast cancer due to its association with abnormal activity of p53 40. SETD2 mutation in osteosarcoma can inhibit the growth of tumor cells. In addition, it significantly affects cisplatin-induced apoptosis, resulting in inactivation of Wnt/β-catenin signaling, and sequentially leading to dysfunction of downstream c-myc, cyclin D1 and CD133 93. Malignant peritoneal mesothelioma is a rare tumor with limited treatment 92,97, gene sequencing of 13 patients with malignant mesothelioma has shown SETD2 mutation in malignant peritoneal mesothelioma, and it may be linked to PI3K-mTOR signaling pathway, which is expected to become a new target for the treatment of this type of tumor 92,97 (Table 2).

## Conclusions

In the present review, we summarized all recent findings on SETD2 mutation or its functional loss in a variety of solid tumors. SETD2 plays an important role in the initiation, progression, prognosis and treatment of solid tumors. SETD2 participates in transcriptional regulation, DNA MMR, human DNA HR and some other processes, leading to progression of malignant tumor. Mutation of SETD2 gene or its functional loss causes dysfunction of downstream signaling pathways, including wnt signaling pathway, PGC1α metabolic pathway and PI3K-mTOR signaling pathway, and such dysfunction is related to tumor occurrence. In addition, there are some signaling pathways, which are linked to drug resistance, such as Wnt/β-catenin signaling pathway and ERK signaling pathway. Taken together, SETD2 may be a regulator of tumor suppressor and can be used as a novel prognostic biomarker and therapeutic target. However, the underlying mechanism remains unknown, and there is still a lack of relevant research, especially in vitro studies. Therefore, it is necessary to clarify how SETD2 is involved in oncogenesis, progression and prognosis of cancers, which may help select the most appropriate and cost-effective therapeutic drugs, and monitor recurrence and drug resistance, and assess prognosis for each patient.

## Figures and Tables

**Table 1:** Overview of SETD2 mutation in a selection of solid tumors based on the COSMIC database (Mar. 21, 2019)

Tissue/tumor subtype	Number and percentage of samples with mutation (%)									Total mutated samples (%)	Total samples tested
	Nonsense substitution	Missense substitution	Synonymous substitution	Inframe insertion	Frameshift insertion	Inframe deletion	Frameshift deletion	Complex mutation	Other		
Kidney	83 (26.52%)	95 (30.35%)	5 (1.60%)	2 (0.64%)	20 (6.39%)	4 (1.28%)	77 (24.60%)	1 (0.32%)	4 (1.28%)	313 (9.19%)	3407
Skin	18 (19.15%)	70 (74.47%)	8 (8.51%)	0 (0.00%)	1 (1.06%)	0 (0.00%)	1 (1.06%)	1 (1.06%)	0 (0.00%)	94 (5.39%)	1744
Not specified (NS)	8 (26.67%)	17 (56.67%)	2 (6.67%)	0 (0.00%)	0 (0.00%)	0 (0.00%)	1 (3.33%)	0 (0.00%)	1 (3.33%)	30 (5.35%)	561
Pleura	10 (40.00%)	6 (24.00%)	0 (0.00%)	0 (0.00%)	0 (0.00%)	0 (0.00%)	9 (36.00%)	0 (0.00%)	0 (0.00%)	25 (5.35%)	467
Large intestine	13 (8.12%)	107(66.88%)	23 (14.37%)	0(0.00%)	3(1.88%)	0(0.00%)	39(24.38%)	0(0.00%)	1(0.62%)	160(4.46%)	3590
Urinary tract	1 (2.08%)	41 (85.42%)	4 (8.33%)	0 (0.00%)	0 (0.00%)	0(0.00%)	3 (6.25%)	0 (0.00%)	0 (0.00%)	48 (3.98%)	1206
Endometrium	5 (14.29%)	31 (88.57%)	5 (14.29%)	0 (0.00%)	0 (0.00%)	0 (0.00%)	2 (5.71%)	0 (0.00%)	0 (0.00%)	35 (3.69%)	948
Lung	36 (23.53%)	90 (58.82%)	5 (3.27%)	0 (0.00%)	8 (5.23%)	1 (0.65%)	15 (9.80%)	3 (1.96%)	0 (0.00%)	153 (3.58%)	4268
Liver	11 (16.18%)	43 (63.24%)	9 (13.24%)	0 (0.00%)	3 (4.41%)	0 (0.00%)	5 (7.35%)	0 (0.00%)	0 (0.00%)	68 (2.95%)	2307
Soft tissue	6 (18.18%)	23 (69.70%)	0 (0.00%)	0 (0.00%)	0 (0.00%)	0 (0.00%)	3 (9.09%)	0 (0.00%)	1 (3.03%)	33 (2.5%)	1320
Breast	22(22.92%)	49(51.04%)	7(7.29%)	0(0.00%)	2(2.08%)	6(6.25%)	19(19.79%)	1(1.04%)	1(1.04%)	96(2.24%)	4278
Stomach	0(0.00%)	17(65.38%)	4(15.38%)	0(0.00%)	0(0.00%)	0(0.00%)	5(19.23%)	0(0.00%)	0(0.00%)	26(2.12%)	1225
Cervix	1(12.50%)	8(100.00%)	0(0.00%)	0(0.00%)	0(0.00%)	0(0.00%)	0(0.00%)	0(0.00%)	0(0.00%)	8(2.01%)	398

*Tumor subtypes with a sample size less than 100 cases and mutation frequencies less than 2% have been excluded.

**Table 2:** Overview of SETD2 in a selection of solid tumors based on this work

Tissue/tumor subtype	Function	Mechanism	Gene change	Reference/s
Clear cell renal cell carcinoma	tumorigenesis	SETD2-PGC1α metabolic pathway	loss	67
	treatment	SETD2-MCL-1 axis	mutation	68
	Prognosis	unknown	mutation	57
Lung cancer	Tumorigenesis/progression	SWI/SNF complex	loss	83, 87
	therapy	ERK signaling pathway	mutation	82
Gastric cancer	proliferation, migration, invasion/ prognosis	p53	Mutation/loss	73-75.
Colorectal cancer	Tumorigenesis/proliferation/metastasis	alternative splicing/ wnt signal pathway	mutation	76, 77
Central nervous system tumors	Tumorigenesis	unknown	mutation	90, 91
Pancreatic cancer	Tumorigenesis	unknown	mutation	8,79
prostate cancer	Treatment	unknown	mutation	72
malignant peritoneal mesothelioma	Tumorigenesis	PI3K-mTOR signal pathway	mutation	92
osteosarcoma	tumorigenesis, treatment	Wnt/β-catenin signal pathway	mutation	93
Breast cancer	Prognosis	p53	mutation	40, 94

## Abbreviations

SETD2: SET domain-containing 2
H3K36: histone H3 lysine 36
H3K36me1: monomethylated histone H3
H3K36me2: dimethylated histone H3
H3K36me3: trimethylated H3K36
RNAPII: RNA polymerase II
RPB1: RNAPII subunit B1
KMTs: histone lysine methyltransferases
NSD: nuclear receptor SET domain-containing
ASH1L: Absent small and homeotic disks protein 1 homolog
NSD1, NSD2, NSD3: Nuclear receptor-binding SET domain-containing proteins 1-3
SMYD2: SET and MYND domain containing 2
AWS: associated with SET
PS: post-SET
CC: Coiled-Coiled
SRI: Set2-Rpb1 interacting
CTD: carboxy-terminal domain
MRG15: MORF-related gene on chromosome 15
PTB: polypyrimidine tract binding protein
DSB: DNA double-strand break
HR: DNA homologous recombination repair
RPA: Replication Protein A
PWWP: Proline-tryptophan-tryptophan-Proline
MMR: mismatch repair
LEDGF: lens epithelium-derived growth factor
CtIP: C-terminal binding protein interacting protein
STAT1: signal transducers and activators of transcription
ccRCC: clear cell renal cell cancer
AS: Alternative splicing
GC: gastric cancer
LUAD: lung adenocarcinoma
NSCLC: non-small cell lung cancer
CRC: colorectal cancer
DVL2: dishevelled segment polarity protein 2
